# Development of Stiff, Tough and Conductive Composites by the Addition of Graphene Nanoplatelets to Polyethersulfone/Epoxy Composites

**DOI:** 10.3390/ma11112137

**Published:** 2018-10-30

**Authors:** Fuzhong Wang, Lawrence T. Drzal

**Affiliations:** 1 Advanced Materials Institute, School of Materials Science and Engineering, Qilu University of Technology (Shandong Academy of Sciences), Jinan 250353, China; 2 Composite Materials and Structures Center, Michigan State University, East Lansing, MI 48824-1226, USA; drzal@egr.msu.edu

**Keywords:** polymers, fracture toughness, mechanical properties, thermal properties

## Abstract

In this study, polyethersulfone (PES) was blended into epoxy resins to improve the fracture toughness of the epoxy resin without loss of mechanical properties, and then two grades of pristine graphene nanoplatelets (GnPs) were separately introduced into the PES/epoxy system to fabricate thermally conductive GnPs/PES/epoxy composites with high toughness as well as high stiffness. It was observed that the addition of GnPs obviously affected the final phase morphology by suppressing the phase separation process of the PES modified epoxy due to the increased viscosity and cure-reaction rate of PES/epoxy. The GnPs with a larger lateral dimension revealed a greater reinforcing effect, and the inclusion of 3 wt % GnPs (~5 μm in diameter) endowed the PES/epoxy matrix with a good thermal conductivity and improved the tensile, flexural, and storage modulus by 27.1%, 17.5%, and 15.6% (at 30 °С), respectively. Meanwhile, the fracture toughness was further enhanced by about 29.5% relative to the PES modified epoxy at the same GnPs concentration. The positive results suggest that the modification of epoxy resins using the PES and GnPs is an attractive approach for fabricating tougher and stiffer epoxy-based nanocomposites with multifunctional properties, which could widen the industrial applications of the epoxy resins.

## 1. Introduction

Epoxy resins have been extensively used in many industrial fields, such as aerospace, automotive materials, adhesives, and electronic devices, owing to their good mechanical properties, excellent manufacturability, and corrosion resistance [1,2]. Unfortunately, the neat epoxy resins are inherently brittle and vulnerable to crack growth because of the high cross-linked density which extremely limited the further use of the epoxies. To improve the toughness of the epoxy resins, many studies have been carried out to toughen the neat epoxy by incorporating soft rubbers or thermoplastic polymers, e.g., polyethersulfone (PES), polystyrene (PSF) and polyethylene (PEI) [3,4,5,6]. The soft rubber is often employed as a toughener for polymers, and it could effectively enhance the fracture resistance. However, the improvement is usually accompanied by significant loss of mechanical and thermal properties of the composites. Moreover, the introduction of soft rubbers with low glass transition temperature (T_g_) also reduced the cross-linking density, leading to a decrease in T_g_ of the modified matrix [7]. By contrast, the fracture toughness of thermoset polymers was usually enhanced without compromising the T_g_ and mechanical properties after the addition of thermoplastic polymers due to their inherently high T_g_ and high modulus.

The PES is a special engineering thermoplastic which possesses a relatively high modulus, mechanical strength, and T_g_ (~225 °С) [8]. The incorporation of PES can improve the toughness of the neat epoxy through phase-separation without degradation of T_g_ and mechanical properties [9]. While the fracture toughness enhancement promoted by PES is relatively lower than that of the most commonly used rubbers like carboy-terminated contractile butane and amigo-terminated contractile-butane [3,10,11]. Therefore, it is necessary to further enhance the toughness of the PES modified epoxy by adding a secondary modifier.

With the development of nanotechnology, many different nano-sized fillers such as clay, cellulose nanofibers, carbon nanotubes, silica, as well as graphene have been added to the polymer-based system to fabricate composites with high fracture toughness [12,13,14,15]. For instance, Zhang et al. [14] employed cellulose nanofibers (CNF) to reinforce polystyrene (PSF)/epoxy composites, and it was noticed that the impact strength was increased by ~49% after the introduction of 0.2 wt % CNF. Arkansas et al. [15] fabricated polyamide/epoxy composites modified by multi-walled carbon nanotubes (MWCNTs), and it revealed that the inclusion of MWCNTs obviously enhanced the tensile strength and toughness of the polyamide/epoxy composite. Among various fillers, the carbonaceous fillers have attracted wide scientific interest not only for ameliorating toughness, but also enhancing the thermal and electrical conductivity [16,17]. Graphene, the thinnest two-dimensional structure material, has been widely explored during the past few years due to its excellent mechanical, thermal, and electrical properties [18,19]. However, the manufacture cost of single layer graphene is pretty high, which restrained its practical applications in the field of polymer composites.

Graphene nanoplatelets (GnPs) are composed of multilayered graphene with 1–10 nm in thickness and 0.3–50 μm in diameter, which can be manufactured on a large scale using a top-down approach [20,21]. The GnPs flakes produced therefrom are cost-effective, making them more favorable for reinforcing polymers than the CNTs. Moreover, compared with the entangled CNTs, the GnPs with a two-dimensional structure can be easier to be dispersed in polymer matrix by using suitable techniques, and the addition of GnPs usually having less of an effect on increasing the viscosity of mixtures than that of the CNTs at the same filler content. Therefore, the GnPs were suitable for creating multifunctional composites due to their good properties and low costs [22,23]. During the past few years, much work has been carried out to introduce GnPs into the epoxy resins to modify the matrix properties including thermal and electrical conductivity [24,25,26,27,28]. In our previous study [27], the modulus of the epoxy was greatly enhanced after the introduction of GnPs (~5 μm in diameter), and the resulting composite is thermally conductive due to the presence of the highly conductive GnPs. Chandrasekaran et al. [28] studied the toughness of epoxies reinforced with 1 wt % GnPs, and the toughness was improved by 25%. The promising outcomes encouraged us to utilize GnPs to modify the PES/epoxy binary system and explore the effects of GnPs on the mechanical performance and thermal properties of PES/epoxy composites.

In this study, we aim to modify the neat epoxy resins by the simultaneous usage of PES and GnPs, and achieve high toughness as well as high stiffness within a good thermally conductive epoxy matrix. Specifically, two types of pristine GnPs with different lateral dimension were separately introduced into the PES/epoxy matrix, and the influence of GnPs on the final morphology, fracture toughness, mechanical, and thermal properties of the epoxy-based multi-phase composites were carefully studied. In addition, the mechanisms of the property enhancements—as well as the interactions among the micro-cracks, GnPs, and the matrix—were explored by characterizing the fractured surfaces of the composites with scanning electron microscope (SEM).

## 2. Experimental

### 2.1. Materials

Two grades of GnPs obtained from XG Sciences, Inc. (Lansing, MI, USA) were employed in this study. The one with a lateral size below 1 μm was referred to as GnP-C750 with a specific surface area of 750 m^2^/g, and one with a size of ~5 μm in the in-plane direction was named GnP-5 having a specific surface area of 150 m^2^/g. The thickness of the GnP-C750 is similar to the GnP-5 with the value of ~5 nm. Epoxy resin (Epon 828) with a viscosity of 110–150 P at 25 °C was provided by Miller-Stephenson Chemical Inc., Sylmar, CA, USA. The curing agent is m-Phenylenediamine (m-PDA) which was ordered from Sigma-Aldrich (MilliporeSigma Corp., Louis, MO, USA). PES powders with a particle size below 120 μm were supplied by Solvay Specialty Polymers Inc., Augusta, GA, USA.

### 2.2. Composites Fabrication

In order to disperse GnPs in epoxy resins, the GnPs as well as epoxy resins were weighed and transferred to a beaker containing acetone, and the mixture was subjected to ultrasonication with a high output power. After that, the solution was heated to about 60 °С to vaporize the solvent. Subsequently, a three-roll mill calendaring was employed to disperse GnPs in the resins, and homogeneous GnPs/epoxy mixtures with different amount of GnPs were produced. The detailed dispersing procedure of GnPs in epoxy resins was described in our previous report [27].

To fabricate GnPs/PES/epoxy ternary composites, the prepared GnPs/epoxy mixture was preheated to 120 °C on a hotplate to reduce the viscosity of the system, followed by adding calculated quantity of PES powders under consistently magnetic stirring. After that, the mixture was heated up to 150 °C to ensure that the PES powers were fully dissolved. After being cooled down, the curing agent m-Phenylenediamine was introduced to the homogeneous suspension. Subsequently, the entire mixture was subjected to high speed sheer mixing for 3 min at 3000 rpm, and then was transferred to a vacuum oven for degassing at 70 °C for 15 min. After that, the GnPs/PES/epoxy compound was poured into preheated molds, followed by curing at 100 °C for 2 h and another 2 h at 125 °C. It is important to note that the concentration of the PES in the composites was fixed at 10 wt %, and GnPs/PES/epoxy nanocomposites with 1 wt % and 3 wt % GnPs were produced. Neat epoxy and PES/epoxy composites were also prepared as reference materials.

### 2.3. Characterization

The morphology of the GnPs, dispersion of GnPs and the fractured surfaces of the composites were observed with an environmental scanning electron microscopy (FE-SEM, Zeiss Ultra 60, Zeiss, Oberkochen, Germany). Transmission electron microscopy (TEM) of GnPs was carried out on a JEM 2100F electron microscope (Japan Electronics Corp., Tokyo, Japan) at 200 kV. Fourier transform infrared spectra (FT-IR) were performed on a Nicolet iS10-FTIR spectrometer (Thermo Fisher Scientific Corp., Waltham, USA) in KBr pellets with a spectral resolution of 4 cm^−1^ in the range of 1000–4000 cm^−1^. A Multilab-2000 XPS spectrometer (Thermo Fisher Scientific Corp., Waltham, MA, USA) was also used to characterize the GnPs.

A UTS SFM-20 machine (United Calibration Corp., Shanghai, China) was utilized to determine the mechanical properties of the prepared specimens at room temperature following ASTM D638 and ASTM D790 standard, respectively. Dynamic mechanical analyzing was carried out on a dynamic mechanical analyzer (Q800, TA Instruments, New Castle, PA, USA) at a frequency of 1 Hz with temperature ranging from room temperature to 200 °C. The thermal diffusivity was collected from a LFA447 Nanoflash (Netzsch, Selb, German) operated at 25 °C. Compact Tension (CT) testing was performed on an instron tensile machine 1185 (Instron, Shanghai, China) to explore the fracture behavior, and the toughness of the prepared specimens was calculated following the ASTM 5045-99 standard.

## 3. Results and Discussion

### 3.1. Characterization of GnPs and Dispersion of GnPs in Matrix

**Figure 1:**
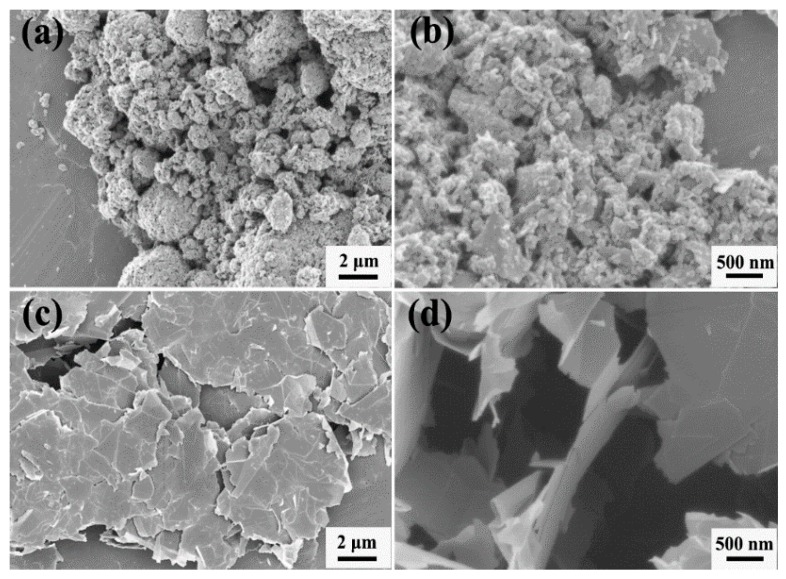
SEM images of (**a**) GnP-C750, (**b**) high magnification of GnP-C750 after sonication, (**c**) GnP-5, (**d**) high magnification of GnP-5 after sonication.

Figure 1 shows the morphology of the GnPs. As seen from Figure 1a, the as-received GnP-C750 is in the form of agglomerates. Figure 1b shows that the average size of GnP-C750 is below 1 μm after sonication. By contrast, the GnP-5 is relatively larger with an average particle size of ~5 µm in the in-plane direction (Figure 1c). Figure 1d shows the GnP-5 after sonication, and the GnP-5 is still in multilayered form.

**Figure 2:**
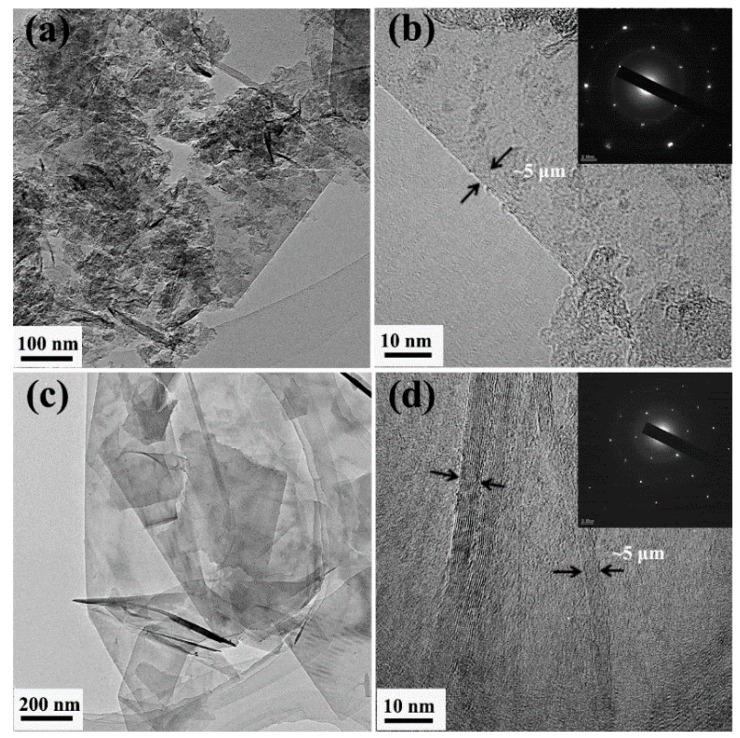
(**a**) TEM image of GnP-C750, (**b**) HRTEM image of GnP-C750, (**c**) TEM image of GnP-5, (**d**) HRTEM image of GnP-5 (insets: electron diffraction patterns).

The GnPs were also characterized with TEM. As illustrated in Figure 2a, the GnP-C750 is tiny, and the high-resolution transmission electron microscopy (HRTEM) image of GnP-C750 (Figure 2b) shows that the thickness of the GnP-C750 is around 5 nm. It is noticed from Figure 2c that the GnP-5 exhibits a larger lateral dimension than the GnP-C750, while the thickness of the GnP-5 is comparable to that of the GnP-C750 (Figure 2d). The electron diffraction pattern of GnPs shown in the inset of Figure 2b,d affirms the structure of multilayered GnPs [29].

**Figure 3:**
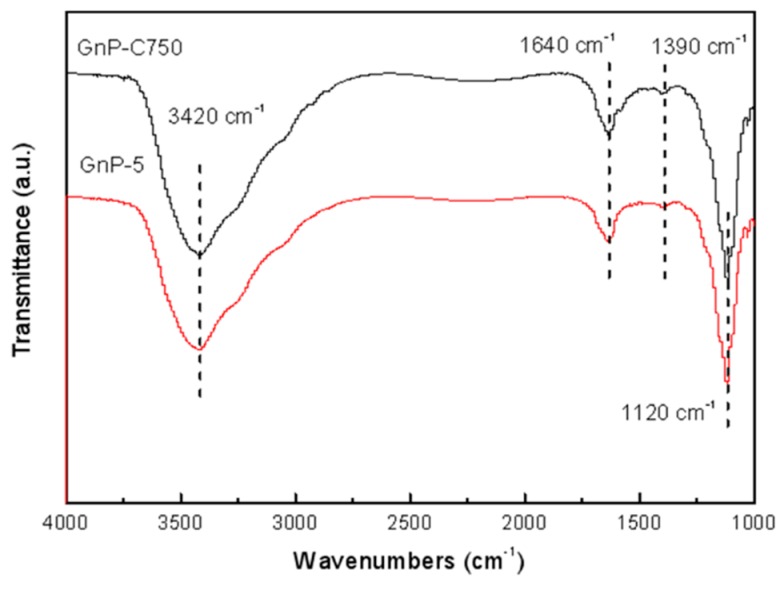
FTIR spectra of GnP-C750 and GnP-5.

FTIR spectra are reported in Figure 3. As can be seen, the wide band centered at 3420 cm^−1^ corresponds to the O-H (hydroxyl groups), which may result from the surface hydroxyls on GnPs or the water that absorbed in KBr pellets. The absorption peak at 1640 cm^−1^ owing to the C=C stretching of the skeletal vibration [30,31]. A small peak was observed at 1390 cm^−1^ which is generally related to the O-H bending stretching vibrations, and the sharp peak at 1120 cm^−1^ can be attributed to the C-O-C (epoxy/ether groups) stretching [32]. The FTIR spectra of the GnPs suggests that the oxygen-containing groups have been included in both GnP-C750 and GnP-5.

**Figure 4:**
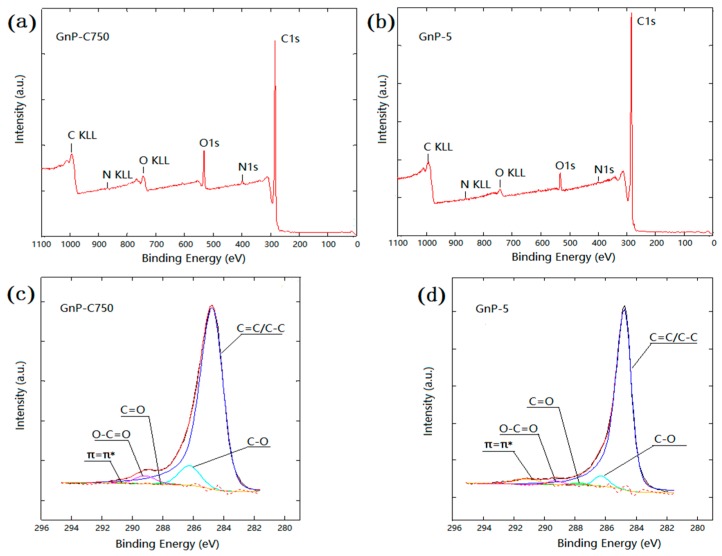
XPS spectra of (**a**) GnP-C750 and (**b**) GnP-5; C1s peak of (**c**) GnP-C750 and (**d**) GnP-5.

Figure 4 shows the XPS spectra of the GnPs, and the atomic concentration were summarized in Table 1. Figure 4a,b indicate that O1s peak for GnP-C750 is stronger as compared to the GnP-5. As seen from Table 1, the oxygen element concentration of GnP-C750 is 8.79% measured by XPS, which is twice higher than the value of GnP-5 (4.01%). The higher O/C ratio of GnP-C750 shown in Table 1 also indicated that there are more oxygen-containing groups on GnP-C750 relative to GnP-5. The deconvolution of the peak related with carbon atoms were presented in Figure 4c,d, and a strong peak positioned at 284.8 eV was observed in the X-ray photoelectron spectroscopy spectrum, which is attributed to the sp^2^ and sp^3^ carbon of the GnPs. Additional signals at 286.3, 287.8, and 289.3 eV also appeared, which correspond to three types of carbon bonds: C-O, C=O, and O-C=O, respectively [11,33].

**Figure 5:**
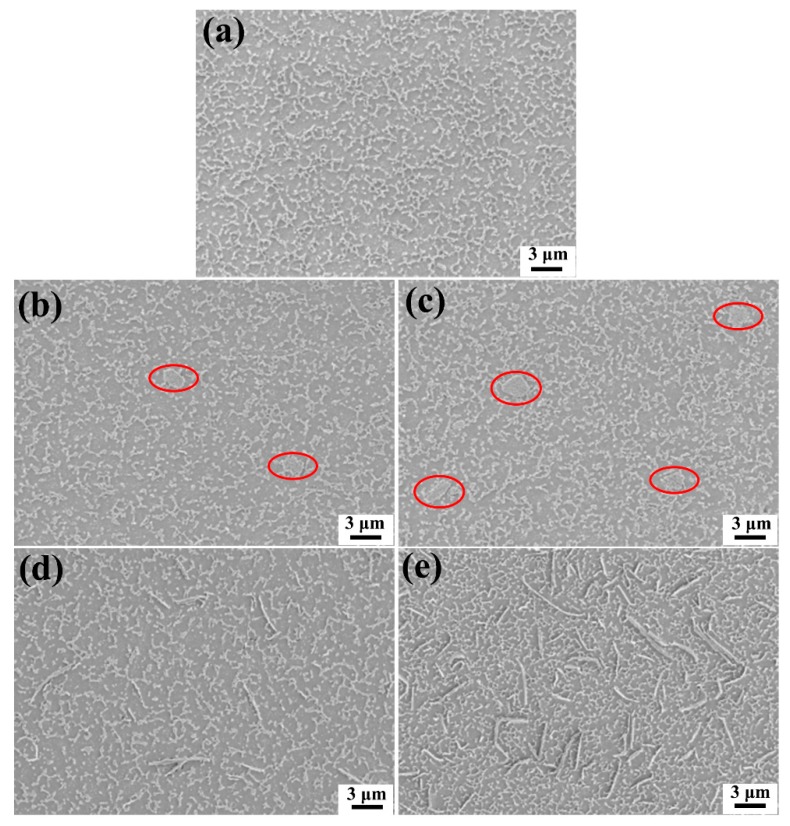
Dispersion of GnPs in PES/epoxy composites:(**a**) without GnPs, (**b**) 1 wt % GnP-C750, (**c**) 3 wt % GnP-C750, (**d**) 1 wt % GnP-5, (**e**) 3 wt % GnP-5.

To analyze the dispersion of the GnPs, the samples were polished to be smooth, and followed by plasma etching. Figure 5a shows the PES/epoxy composites without GnPs, and it is apparent that the thermoplastic PES was etched and the particles were distributed on the surface of the sample. Figure 5b,c illustrate the distribution of GnP-C750 in composites reinforced with 1 wt % and 3 wt % fillers, respectively. Agglomerates of GnP-C750 with a size below 3 μm appeared in the composites as indicated by the red ellipse frames shown in Figure 5b,c. Compared with our previous study where the neat epoxy was only modified with GnP-C750 [27], the GnP-C750 is more difficult to be dispersed in the PES/epoxy system due to the extremely high viscosity of the PES/epoxy mixture. By contrast, it was found that the GnP-5 particles are well dispersed at various concentrations as manifested in Figure 5d,e, respectively.

### 3.2. Tensile and Flexural Properties

**Figure 6:**
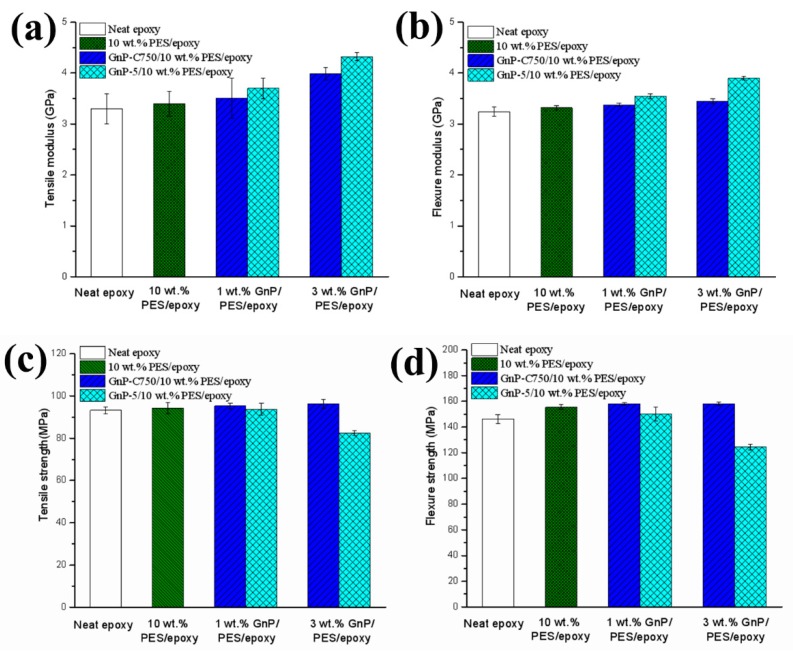
(**a**) Tensile modulus, (**b**) flexural modulus, (**c**) tensile strength, and (**d**) flexural strength of epoxy-based composites.

Figure 6 illustrates the mechanical properties of the epoxy-based composites. The addition of 10 wt % PES slightly enhanced the tensile and flexural properties (modulus and strength) of the epoxy [34]. After the PES/epoxy composites were further modified with GnPs, it is noticed from Figure 6a,b that the modulus of the GnPs/PES/epoxy composites experienced an increasing trend regardless of the particle size of GnPs. However, the GnP-5 flakes revealed a more pronounced improvement in the modulus thanks to the larger lateral dimension of GnP-5 that uniformly distributed in matrix. The tensile and flexural modulus of the PES/epoxy hybrid sample were increased by 27.1% and 17.5% respectively after the inclusion of 3 wt % GnP-5. It is worth mentioning that the GnPs/PES/epoxy composite is much stiffer as compared to the organoclay/PES/epoxy and GnPs/CTBN/epoxy composites reported previously [11,12]. For the strength, it can be seen from Figure 6c,d that the mechanical strength of the composites reinforced with GnP-C750 are comparable to that of the PES modified epoxy or neat epoxy. For the PES/epoxy composites reinforced with GnP-5, no significant change in mechanical strength was observed when 1 wt % GnP-5 was incorporated, while the tensile and flexural strength decreased to some extent after the introduction of 3 wt % GnP-5. To explore the reasons of the above-mentioned phenomena, the SEM was utilized to characterize the fractured surfaces of composites and the analyses were presented in the following paragraph.

**Figure 7:**
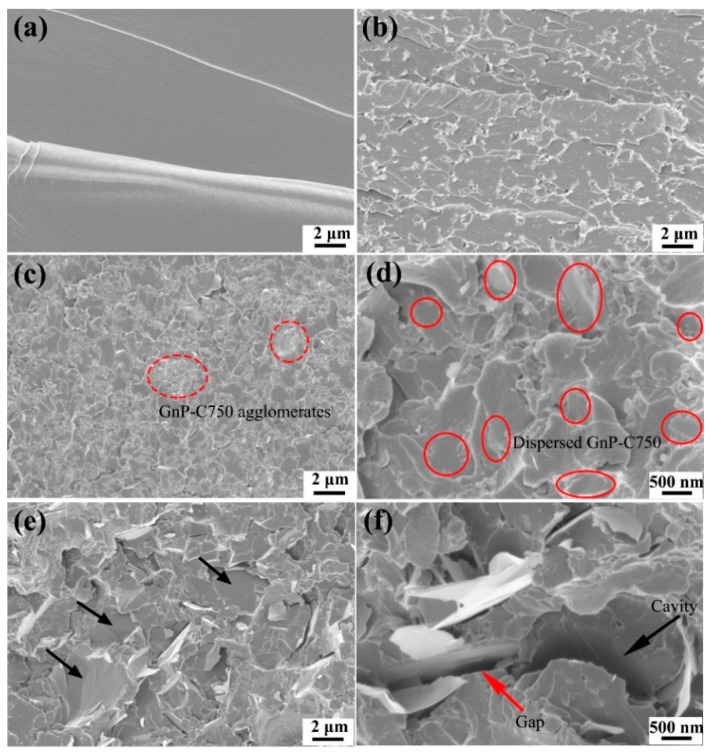
SEM images of the tensile fractured surfaces of (**a**) neat epoxy, (**b**) PES/epoxy, (**c**) 3 wt % GnP-C750/PES/epoxy, (**d**) 3 wt % GnP-C750/PES/epoxy (magnified), (**e**) 3 wt % GnP-5/PES/epoxy, (**f**) 3 wt % GnP-5/PES/epoxy (magnified).

Figure 7 shows SEM images of fracture surface of the epoxy-based composites obtained from tensile testing. As illustrated in Figure 7a, the fractured surface of the unmodified epoxy reveals a relatively plane and glassy microstructure. By contrast, the epoxy composite modified with 10 wt % PES exhibits a particulate morphology, and the PES particles are uniformly distributed in the continuous epoxy matrix (Figure 7b). For the GnP-C750 reinforced composites, agglomerates were observed as shown in Figure 7c (red dashed ellipse frames), a larger magnification presented in Figure 7d shows that dispersed GnP-C750 are well embedded in the matrix (red ellipse frames). Therefore, the good GnP-C750/matrix interfacial adhesion of the GnP-C750/PES/epoxy composites contributed to a comparable composite strength with PES/epoxy binary composites despite the presence of small GnP-C750 agglomerates in composites. Compared with the GnP-C750 case, the fracture surface of the GnP-5 reinforced composites is relatively rougher, and the GnP-5 particles are uniformly distributed in the composites as shown in Figure 7e. It was also observed from Figure 7e that GnP-5 particles with smooth and clean surface were detected as indicated by the black arrows, indicating poor interfacial adhesion between the GnP-5 and matrix. A magnified image provided in Figure 7f shows a clear gap between the GnP-5 and matrix (the red arrow) as well as a quite large cavity that was induced by the pull-out of GnP-5 during fracture (the black arrow). The observed phenomena suggest that the GnP-5/matrix interfacial adhesion is much weaker as compared to that of the GnP-C750 case, and this might be resulted from the absence of functional groups of GnP-5 as detected in Figure 4 and Table 1. The weak GnP-5/matrix interfacial interactions make GnP-5 serve as impurities and promote catastrophic failure of composites under loading stress, leading to the decline of the mechanical strength as reported in Figure 6c,d.

### 3.3. Dynamic Mechanical Properties

**Figure 8:**
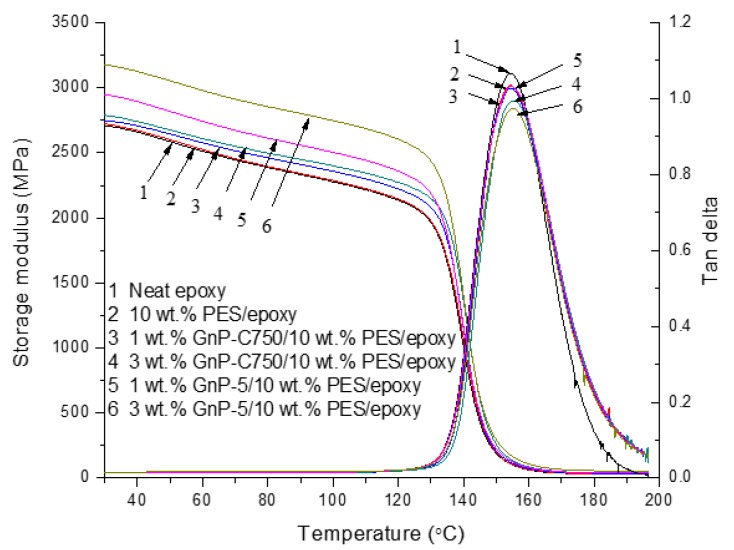
Dynamic mechanical properties of epoxy-based composites.

The dynamic mechanical properties of the cured specimens is displayed in Figure 8. The storage modulus at 30 °C and T_g_s of the prepared samples were collected and are presented in Table 2. After the epoxy was modified with 10 wt % PES, there is no significant change in storage modulus with temperature ranging from 30 °C to 200 °C (curves 1 and 2). For the ternary composites, it is noticeable from curves 3 and 4 that the storage modulus of PES/epoxy was enhanced after the inclusion of the multilayered graphene, and the value at 30 °C was increased from 2.72 to 2.75 and 2.79 GPa after the addition of 1 and 3 wt % GnP-C750, respectively. A notable improvement for the GnP-5 case was observed, and the storage modulus at 30 °C reached 2.95 and 3.18 GPa at 1 wt % and 3 wt % GnP-5, corresponding to 8.5% and 15.6% enhancements relative to the PES modified epoxy, respectively. The obvious enhancements of storage modulus after the introduction of GnPs with larger particle size may result from the good dispersion and large lateral dimension of GnP-5 which induced strong interactions between the GnP-5 and epoxy molecules. Figure 8 also presents the variation of loss tangent of the prepared samples. For the neat epoxy, a peak value of tan δ appeared in curve 1 at 154.3 °C, corresponding to the T_g_ of epoxy. After the addition of PES, the T_g_ of the composites does not change much, and a single tan delta peak appeared for the PES modified matrix resins (curve 2), suggesting that the PES particles are uniformly distributed in the epoxy. After the PES/epoxy composites were further modified with GnPs, the GnPs/PES/epoxy composites were found to exhibit a slightly higher T_g_ as recorded in Table 2. This could be attributed to the restriction of the mobility of epoxy segments induced by the dispersed GnPs through interfacial interactions.

### 3.4. Thermal Conductivity

**Figure 9:**
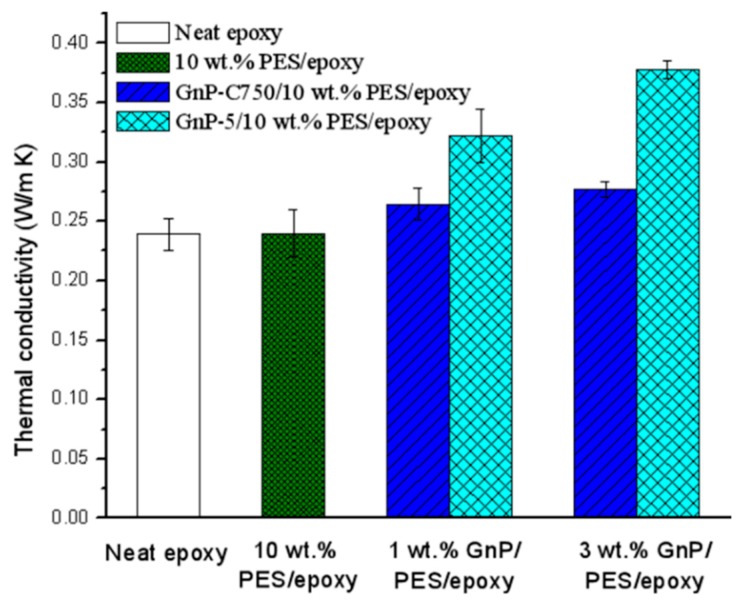
Thermal conductivity of epoxy-based composites.

The thermal conductivity of the prepared composites was characterized and is shown in Figure 9. The conductivity of the unmodified epoxy is about 0.23 W/m·K, and the value does not show any change after the introduction of 10 wt % PES. For the GnPs/PES/epoxy ternary composites, the value notably increased with the increases of filler content as expected due to the inclusion of the high thermally conductive GnPs. The incorporation of 3 wt % GnP-C750 and GnP-5 resulted in higher thermal conductivities, reaching 0.28 W/m·K and 0.38 W/m·K with enhancements of 21.7% and 65.2%, respectively. Obviously, it is noticed from Figure 9 that the GnP-5 performed better than GnP-C750 in enhancing the conductivity of the composites, and the result is in good agreement with Xiao’s work [35] where a special computational model was created for evaluating the thermal behavior of the GnPs/epoxy composites. The better performance of GnP-5 is probably ascribed to two possible factors. First, it is well known that efficient heat propagation in composites is largely ascribed to diffusion of phonons [36]. As seen from Figure 5, compared with GnP-C750, the GnP-5 with a larger aspect ratio showed a better distribution in the composites without any agglomerates, and this facilitates GnP-5 particles to form more efficient thermally conductive networks that are preferable for phonon diffusion. Second, considering the large lateral dimension of GnP-5, there would be less particle/particle and particle/matrix nano-interfaces within the GnP-5 reinforced PES/epoxy composites than the GnP-C750 case [37]. As a result, the lower contact resistance of interfaces and less phonon scattering at the boundaries of the GnP-5/PES/epoxy composites promoted a higher conductivity relative to the GnP-C750 case.

### 3.5. Fracture Toughness, Morphology, and Failure Mechanisms

**Figure 10:**
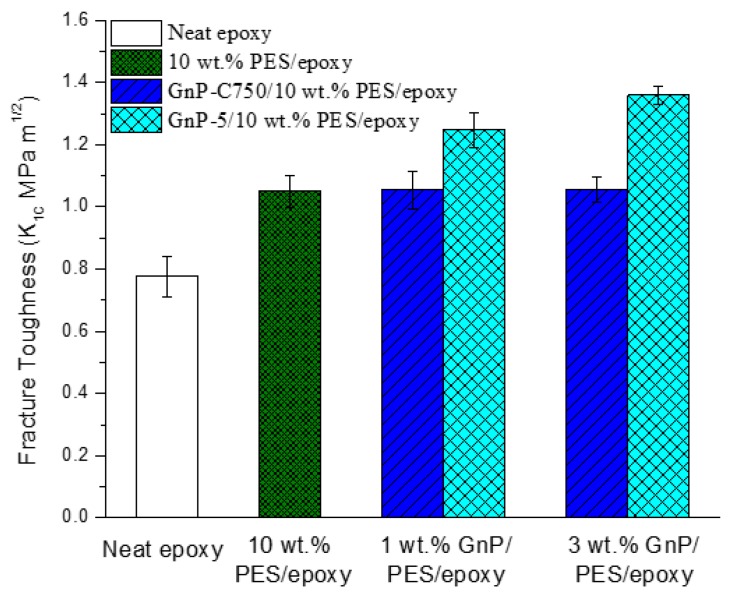
Fracture toughness of epoxy-based composites.

The toughness of the prepared samples were collected and the data are provided in Figure 10. The unmodified epoxy exhibits a low value in fracture toughness, and the value was increased to 1.05 MPa m^1/2^ after modified with 10 wt % PES owing to the phase-separation structure formed. We also observed that there was no obvious change in fracture toughness after the GnP-C750 was introduced into the PES/epoxy composites. By contrast, the toughness value of the PES/epoxy composites was obviously improved after reinforced with GnP-5, reaching 1.36 MPa m^1/2^ at 3 wt % GnP-5, representing about 29.5% and 74.4% enhancements compared with the PES/epoxy binary and the neat epoxy, respectively. The study suggests that a good balance between the enhancement of toughness and stiffness can be achieved by using GnP-5. A comparison with other fillers is shown in Table 3.

**Figure 11:**
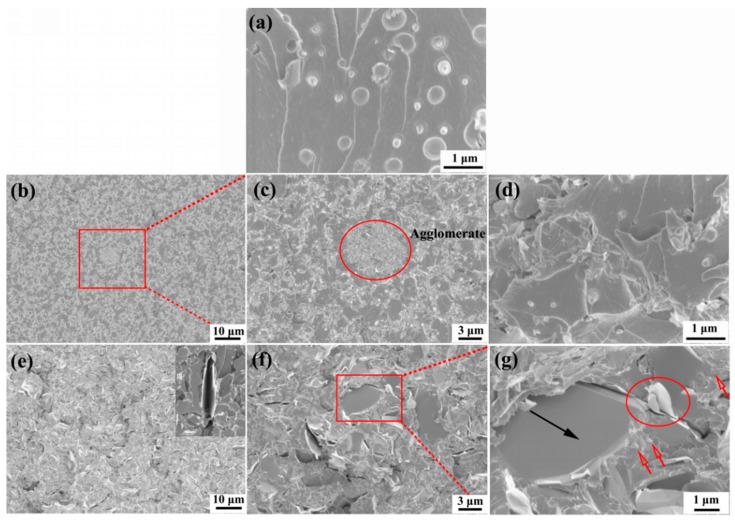
SEM images of CT fracture surfaces of GnPs/PES/epoxy composite: (**a**) 0 wt % GnPs; (**b**–**d**) 3 wt % GnP-C750; (**e**–**g**) 3 wt % GnP-5.

The fractured surfaces were analyzed with SEM after CT testing to explore the modifying mechanisms. The SEM image for neat epoxy was not provided because the pure epoxy usually exhibits a flat and featureless fracture surface [40]. After the epoxy was incorporated with 10 wt % PES, a sea-island microstructure was detected because of phase separation of the mixed system. According to results reported in literature [41,42], the phase separation usually follows nucleation growth mechanism at low PES content during the curing process. As the cure-reaction goes on, the PES starts the process of evolution and grows larger, as a consequence, the composites reveals a heterogeneous morphology, and the PES particles are uniformly distributed in the epoxy matrix as shown in Figure 11a. It is usually considered that the toughness enhancement of the PES modified epoxy composite is mainly ascribed to shear yielding of matrix and crack pinning of PES that restrict the initiation of micro-cracks during the toughness testing [4,43]. Figure 11b shows the PES/epoxy composites containing 3 wt % GnP-C750, and the agglomeration of GnP-C750 was clearly detected as indicated by the red ellipse frame in a high-resolution image (Figure 11c). The SEM image shown in Figure 11d with the same magnification as Figure 11a displays a relatively rougher and irregular surface, and this is attributed to the local plastic deformation induced by the interactions between cracks and the small dispersed GnP-C750 during fracture. The plastic deformation was usually considered as one of the toughening mechanisms in composites. However, in the present study, the toughening effect caused by the plastic deformation was offset by the GnP-C750 agglomerates in the rigid PES/epoxy matrix, and the GnP-C750/PES/epoxy composites revealed a comparable fracture toughness with that of the PES/epoxy composites without GnP-C750. In addition, it is worthwhile to mention that, as the viscosity of epoxy system increases, the phase separation rate slowed down as the molecular chains are not able to move freely. It is clearly observed from Figure 11d that the PES was smaller than that in the PES modified epoxy composites. This was attributed to the suppression effect of GnP-C750 on the evolution of PES particles. For one thing, the incorporation of GnP-C750 largely increased the viscosity of the system, which may have prevented the nucleation and growth of PES in the composites [41,42]. As a result, the rate of the PES evolution became slow and the phase separation process was delayed, resulting in small PES particles in the final composites. The presence of GnPs may serve as catalyzer and accelerate the curing process of the matrix, and shorter times are available for the PES to grow before cured, resulting in smaller PES particles as compared to that of the PES/epoxy binary system.

For the GnP-5/PES/epoxy ternary composites, as seen from Figure 11e,f, the fractured surface is much rougher than that of the GnP-C750 case due to the crack deflection of GnP-5. The deflection effect of GnP-5 resulted in a larger crack area which dissipated a great deal of fracture energy and contributed to enhancements of the toughness [28,44]. Besides the deflection effect, separation or delamination in-between GnP-5 flakes may also occur during the fracture process (see the inset in Figure 11e) if the GnP-5 perpendicularly or parallelly oriented to the crack direction. The separation/delamination of GnP-5 layers can also consume energy during the sample failure and was commonly observed in composites reported elsewhere [28,45]. The GnP-5 possesses a relatively lower specific surface area than the GnP-C750, and was found to be uniformly distributed in the fractured surface without any agglomerates as indicated in Figure 11f. A typical magnified SEM image was shown in Figure 11g, and the high-resolution image shows that the crack path was deflected by the micro-sized GnP-5 as indicated by the black arrow. We also observed that layer breakage of GnP-5 also happened as shown by the red ellipse frame, which was also believed to dissipate energy. However, the suppression effect of GnP-5 resulted in tiny and limited amount of PES particles dispersed in the fractured surface as indicated by the red arrows. Based on the analyses of the fracture morphology, it is suggested that the toughening effect of the PES was weakened in the ternary composites own to the suppression effect of GnP-5 on the evolution of PES and coverage effect of GnP-5 on PES particles along the fractured surface. The crack deflection, separation/delamination, and the layer breakage of the GnP-5 layers are believed to mainly contribute to the good fracture behavior of the GnP-5/PES/epoxy multi-phase composites.

**Figure 12:**
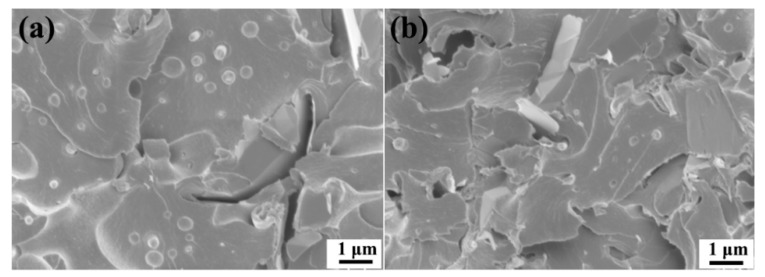
SEM images of CT fracture surfaces of GnP-5/PES/epoxy composite: (**a**) 1 wt % GnP-5 and (**b**) 3 wt % GnP-5.

The fracture morphology of the GnPs reinforced polymer composites is largely determined by the content of the GnPs. Figure 12a,b show the fracture surfaces of the GnP-5/PES/epoxy composites reinforced with 1 wt % and 3 wt % GnP-5, respectively. As can be seen, the PES particles are randomly dispersed in the fractured surfaces, while the PES particle size in the 3 wt % GnP-5 case is obviously smaller than that in composites with 1 wt % GnP-5. This suggests that the higher filler content can lead to greater suppression effect on the phase separation due to the higher viscosity of the mixture and faster curing rate of the reaction system [41]. Besides, the 3 wt % GnP-5 composite exhibits a rougher fracture surface as compared to that of the 1 wt % GnP-5 case, indicating more energy was absorbed during fracture, which is in accordance with the toughness data shown in Figure 10. 

## 4. Conclusions

In summary, the GnPs were successfully introduced into the PES/epoxy composites, and thermally conductive GnPs/PES/epoxy ternary composites with high toughness as well as high stiffness were achieved. SEM observations revealed that a good dispersion of GnP-5 flakes in composites was obtained, while agglomerates of GnP-C750 appeared in the GnP-C750 reinforced composites. It was noticed that the addition of GnPs obviously suppressed the phase-separation of the PES/epoxy mixture and subsequently influenced the final sample morphology. The analyses of the mechanical and thermal properties suggest that the incorporation of GnP-5 into the PES modified epoxy produced greater enhancements in thermal conductivity as well as the tensile, flexural, and storage modulus compared to the GnP-C750 due to the larger lateral dimension and better distribution of GnP-5. Furthermore, the addition to GnP-5 further enhancing the toughness of the PES modified epoxy, which mainly results from crack deflection, separation/delamination, and layer breakage of the GnP-5 layers.

## Figures and Tables

**Table 1:** Atomic concentration of GnPs collected from XPS results

Element Atomic (%)	C (%)	O (%)	N (%)	O/C (%)
GnP-C750	89.48	8.79	1.73	0.0982
GnP-5	95.82	4.01	0.17	0.0418

**Table 2:** Storage modulus and T_g_ of the epoxy-based composites

Specimen	Neat Epoxy	PES/Epoxy	GnP-C750/PES/Epoxy		GnP-5/PES/Epoxy	
GnPs content	0 wt %	0 wt %	1 wt %	3 wt %	1 wt %	3 wt %
Storage modulus at 30 °C (GPa)	2.71	2.72	2.75	2.79	2.95	3.18
T_g_ (°C)	154.3	154.6	154.7	155.3	154.8	155.5

**Table 3:** Comparison of other fillers for thermoplastic polymer modified epoxy.

Filler Fraction	Modified Epoxy	Toughness (MPa m^1/2^) (Enhancements)	Tensile Modulus (GPa) (Enhancements)	Reference
MWCNTs (0.5 wt %)	PES (10.6 wt %)/epoxy	2.02 (10.1%)	4.80 (13.5%)	[38]
MWCNTs (1 wt %)	Polyamide (20 wt %)/epoxy	1.10 (29.4%)	2.38 (−0.4%)	[1]
Organoclay (1 wt %)	PES (5 wt %)/epoxy	1.12 (43.6%)	2.98 (3.5%)	[39]
GnP-5 (3 wt %)	PES (10 wt %)/epoxy	1.36 (29.5%)	4.31 (27.1%)	This work
